# Taurine Facilitates the Formation of Hepatocellular Carcinoma via the Bile Acid Pathway

**DOI:** 10.3390/metabo16010006

**Published:** 2025-12-22

**Authors:** Qin Huang, Xianjiao Mao, Tian Zhang, Yiwen Zhang, Zhaoshuang Lan, Rong Fang, Jiaqi Xiong, Jiahao Li, Yue Sun

**Affiliations:** Yunnan Key Laboratory of Cell Metabolism and Diseases, School of Life Sciences, Yunnan University, Kunming 650500, China; huangqin_zxo0@stu.ynu.edu.cn (Q.H.); maoxianjiao@stu.ynu.edu.cn (X.M.); zhangtian@stu.ynu.edu.cn (T.Z.); zhangyiwen@stu.ynu.edu.cn (Y.Z.); lanzhaoshuang@stu.ynu.edu.cn (Z.L.); 12024114131@stu.ynu.edu.cn (R.F.); 12024214007@stu.ynu.edu.cn (J.X.); 12024114130@stu.ynu.edu.cn (J.L.)

**Keywords:** hepatocellular carcinoma, taurine, bile acids

## Abstract

Backgrounds: While the conditionally essential amino acid taurine is known to confer hepatoprotection against injury through anti-inflammatory and antioxidant mechanisms, it remains unclear whether it plays an active role in the process of hepatocarcinogenesis. Emerging research portrays taurine as a double-edged sword in oncology, with its capacity to either inhibit or facilitate carcinogenesis being contingent upon the specific tumor microenvironment. Objectives: Investigating the effect of taurine on hepatocellular carcinoma progression and its underlying mechanisms. Methods: A hydrodynamic tail vein injection (HDT) model of primary hepatocellular carcinoma was established in mice to validate the effects of taurine and its downstream bile acid synthesis pathway on liver cancer progression. Subsequent RNA sequencing analysis was performed to investigate the molecular pathways through which taurine exerts its functions. Results: Supplementation of taurine or overexpression of its transporter SLC6A6 significantly accelerated HCC development in vivo. Inhibition of taurine transporter abrogated the tumor-promoting effects of the bile acid synthesis enzymes CYP7A1 and BAAT. This suppression may be mediated through the blockade of the cell cycle, p53 signaling pathway and metabolic pathways. Conclusions: Our findings demonstrate that taurine plays a vital role in the tumor-promoting activities of HCC.

## 1. Introduction

Hepatocellular carcinoma (HCC) is the third leading cause of cancer-related death worldwide, accounting for 85% of primary liver cancers [1]. The high mortality rates of HCC make it a significant global public health challenge. Hyperactivation of the WNT/β-catenin pathway is a major contributor to the HCC, as one of the target genes of the WNT/β-catenin signaling pathway, MYC is aberrantly activated in patients with HCC [2,3]. Notably, MYC increases the expression of the taurine transporters SLC6A6 to promote cell growth [4].

Taurine, a conditionally essential amino acid, can be synthesized endogenously, but its primary source is dietary intake (such as meat, seafood, dairy products, and energy drinks), which is facilitated by the high-affinity taurine transporter (SLC6A6) [4,5]. Taurine exhibits numerous critical biological functions. As an intracellular organic osmolyte, Taurine helps maintain cell volume in response to osmotic fluctuations [6]. It also possesses antioxidant and membrane-stabilizing properties and contributes to ion dynamics and calcium homeostasis, particularly in excitable tissues such as the heart [7,8]. Moreover, taurine is indispensable for normal retinal development, as both animal and clinical studies have shown that taurine supplementation contributes to the improvement of retinal abnormalities [9,10,11]. In addition, taurine serves as a key regulator in various physiological and pathological contexts, including aging [12,13], obesity [14], development [15,16], and cardiovascular diseases [17]. In particular, taurine has been linked to cancers [4,18,19]. However, it exerts paradoxical roles in different cancers, inhibiting or promoting carcinogenesis in a context-dependent manner. In gastric cancer, tumor cells outcompete CD8^+^ T cells for taurine. The deficiency of taurine in CD8^+^ T cells induces T cell death and dysfunction, thereby fueling tumor aggressiveness [20]. In leukaemias, elevated expression of SLC6A6 promotes myeloid leukaemia progression by mTOR activation and downstream glycolysis [4]. In the liver, the primary role of taurine is to serve as a substrate for Bile acid (BA) synthesis and to exert a protective effect against CCl4-induced liver damage [21]. However, the role of taurine in liver cancer remains unclear.

BAs are steroid molecules synthesized from cholesterol in the liver. BA biosynthesis is initiated by cholesterol 7α-hydroxylase (CYP7A1). The products are the primary BAs. The primary BAs are conjugated with taurine or glycine by bile acid-CoA: amino acid N-acyltransferase (BAAT) to form bile salts [22]. Notably, liver cancers often exhibit elevated levels of BAs, and increasing hepatic BAs synthesis has been shown to suppress T cell function [23,24]. However, it remains unclear how bile acids affect hepatocytes.

In this study, we found that taurine levels increased in human liver cancer tissues and demonstrated that taurine promotes the formation of HCC via enhancing BAs synthesis. Our work may provide new insights into the pathogenesis of HCC and offer novel avenues for therapeutic intervention.

## 2. Materials and Methods

### 2.1. Mice

All the experimental mice used in this study were managed in accordance with the feeding guidelines of the Experimental Animal Center of Yunnan University, maintained in a Specific pathogen-free (SPF) environment, and fed with standard feed. The FVB mice were purchased from the Experimental Animal Center of Yunnan University. All the experimental animals used were 6-to-8-week-old mice. Therefore, all animal experiments have been approved by the Ethics Committee of the Experimental Animal Center of Yunnan University. This study complied with all relevant ethical regulations. Animal experiments were approved by the Institutional Animal Care and Use Committee of Yunnan University (Approval No. YNU20240705).

### 2.2. Cell Culture

In this study, the human hepatocellular carcinoma cell line HepG2 (American Type Culture Collection, ATCC, Manassas, VA, USA) was utilized. To investigate the effect of taurine on human hepatocellular carcinoma cells, taurine (10 μM) or normal saline was added to the culture medium of HepG2 cells, respectively. Cells were cultured in Dulbecco’s Modified Eagle Medium (DMEM) supplemented with 10% normal or dialyzed fetal bovine serum (FBS, Servicebio, Wuhan, China), glutamine, and antibiotics, and maintained in a 5% CO_2_ incubator at 37 °C. All cell lines were authenticated by short tandem repeat (STR) analysis, examined for their morphological characteristics, and confirmed to be free of mycoplasma contamination. Consistent with the aforementioned experiment, to verify the effect of the taurine transporter inhibitor on human hepatocellular carcinoma cells, HepG2 cells were treated with a taurine transporter inhibitor (0.5 mM) or double-distilled water (ddH_2_O), respectively. The effects of taurine and the inhibitor on human hepatocellular carcinoma cells were evaluated by colony counting and analysis of colony formation.

### 2.3. HDT and Related Experiments

SB100, pT2-shp53 transposon plasmids were obtained from Xin Chen at UCSF. All FVB mice were injected at 6–8 weeks of age, when their body weights were at least 18 g. HDT plasmids were suspended in saline at a volume equivalent to 10% body weight and administered via tail vein injection over 5–7 s. A 10:1 mass ratio of combined HDT plasmids to SB100 transposase plasmid was used. For the experiment to observe the effect of taurine on liver cancer in mice, the above-mentioned cancer plasmid was first injected into 6-week-old FVB male mice. Taurine (Sigma, St. Louis, MO, USA, T0625) was dissolved in saline to prepare a 100 mg/mL stock solution, which was diluted 10-fold prior to intraperitoneal injection into mice at a dose of 100 mg/kg body weight. Then, half of the mice were injected with taurine (every two days) peritoneally, and the other half were injected with normal saline peritoneally. In the in vivo Slc6a6 inhibition experiment, the above-mentioned cancer plasmid was injected into 6-week-old FVB mice, and then half of the mice were peritoneally injected with ddH_2_O or taurine inhibitors (MCE, Monmouth Junction, NJ, USA, HY-113329, 200 mg/kg, every two days).

### 2.4. RNA-Seq

Total RNA was extracted from liver tissues using Trizol, with three mouse samples in each group of experiments, totaling nine samples. Transcriptome sequencing was performed using the Illumina platform to obtain double-ended sequencing data. The RNA-seq library was generated and sequenced by Bioprofile Biotechnology Co., Ltd. (Shanghai, China). Functional enrichment analysis was carried out using clusterProfiler (v4.14.6) for Gene Ontology (GO) and Kyoto Encyclopedia of Genes and Genomes (KEGG) pathway analyses.

### 2.5. RT-qPCR Detection

Total RNA was extracted from liver tissues and then reverse-transcribed into cDNA using an RT kit (AG, Changsha, China). qPCR was performed with SYBR Green qPCR premix kit on a CFX96 real-time PCR detection system (Bio-Rad, Hercules, CA, USA). The data were normalized to the expression level of β-actin. The qPCR primers used in this study, Slc6a6-F: ATGATTGGCTATCGGCCTGG, Slc6a6-R: ACACAGCATGGAGGAAAGGG, β-Actin-F: GTGACGTTGACATCCGTAAAGA, β-Actin-R: GCCGGACTCATCGTACTCC.

### 2.6. AAV Virus Purification

Plasmids encoding target gene overexpression constructed on AAV vectors, along with helper plasmids and viral plasmids, were co-transfected into AAV-293 cells. Chloroform and 5 M NaCl were added for initial viral extraction, followed by centrifugation at 3000 g for 5 min at 4 °C to collect the supernatant. PEG8000 at a concentration of 40% was added to the supernatant, and the viral pellet was collected by centrifugation at 3000 g for 30 min at 4 °C. HEPES buffer, RNase A (TransGen Biotech, Beijing, China, GE101-01), and DNase I (TransGen Biotech, GD201-01) were added, and the virus was purified via chloroform extraction. Concentration and buffer exchange to PBS were performed using a Merck Millipore ultrafiltration tube (100 kDa molecular weight cutoff). AAV was administered into the mouse liver via retro-orbital injection (ROI).

### 2.7. Total Bile Acid (TBA) Assay

Mice injected with bile acid-related plasmids were subjected to eyeball blood collection into coagulant tubes before liver sample harvesting. The blood samples were centrifuged at 3000 rpm at 4 °C for 30 min, and the supernatant was collected as serum. The total Bile Acid Colorimetric Assay Kit (Elabscience, Wuhan, China, E-BC-K181-M) was used to determine the total bile acid content, including both conjugated and non-conjugated forms.

### 2.8. Western Blot

Total protein lysate was extracted by radioimmunoprecipitation assay (RIPA) lysis buffer supplemented with a protease inhibitor (PMSF). Equal amounts of protein were separated by SDS-PAGE gel electrophoresis and then transferred onto a PVDF membrane. The membrane was incubated in a primary antibody solution diluted according to the manufacturer’s recommendations. After hybridization with a secondary antibody conjugated to HRP, protein bands were visualized using a chemiluminescence detection kit on the Bio-Rad ChemiDoc XRS+ imaging system. The antibodies used in this study were as follows: HA (Abcam, Cambridge, UK, ab9110), GAPDH (Bioss, Beijing, China, bsm-0978M), and Flag (Abclonal, Wuhan, China, AE024).

### 2.9. Statistics

All statistical analyses were performed using more than 3 independent biological replicates, or mouse studies were conducted using a specified number of animals. GraphPad Prism 9.3.0 was used for data analysis and graphing, and all summarized data were presented as mean ± standard deviation (SD). For comparisons between 2 groups, an unpaired two-tailed *t*-test was applied. For comparisons among more than 2 groups, one-way analysis of variance (ANOVA) followed by Tukey’s multiple comparison test was used. Results were considered statistically significant when *p* < 0.05, with significance levels denoted as: * *p* < 0.05, ** *p* < 0.01, *** *p* < 0.001, **** *p* < 0.0001. ns indicates no significance.

## 3. Results

### 3.1. Taurine Is Elevated in Liver Cancer and Promotes Human HCC Cell Proliferation in Human HCC Cell Lines HepG2

**Figure 1:**
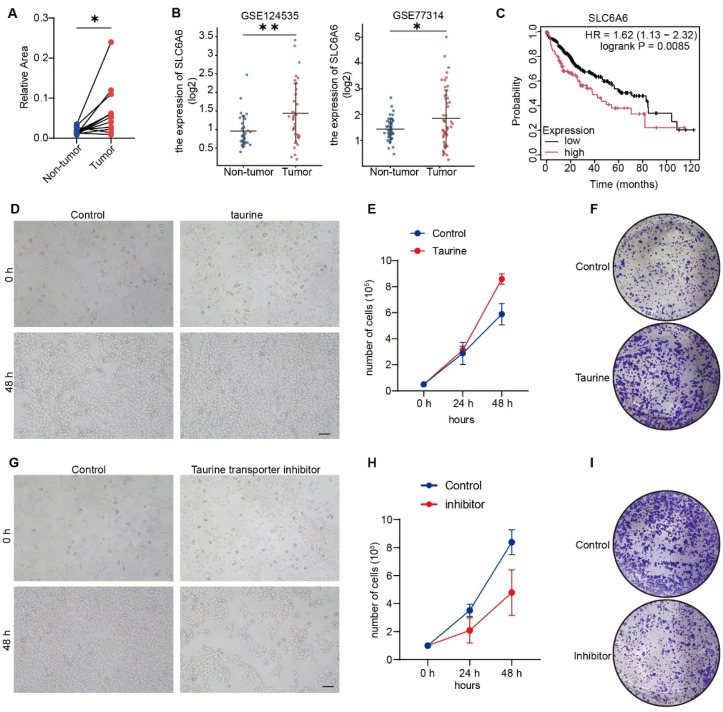
Taurine levels are elevated in liver cancer and promote human HCC cell proliferation. (**A**) Taurine levels in human liver cancer tissues versus normal tissues from metabolomics databases (*n* = 12). (**B**) Analysis of *SLC6A6* mRNA expression in human tumor versus adjacent non-tumor tissues using GEO datasets GSE124535 and GSE77314. (**C**) The Kaplan–Meier plotter analysis shows the survival curves for HCC patients, stratified by high (red) versus low (black) tumor expression of *SLC6A6*. (**D**) The growth status of HepG2 under control and taurine conditions. Scale bars, 100 μm. (**E**) Number of cells demonstrating increased cell proliferation in taurine-treated cells. (**F**) Colony formation assays showed that the colony-forming ability of cells was enhanced after taurine treatment. Representative images and quantification from three independent experiments are included. (**G**) The growth status of HepG2 under control and taurocyamine conditions. Scale bars, 100 μm. (**H**) Number of cells demonstrating decreased cell proliferation in taurocyamine-treated cells. (**I**) Colony formation assays showed that the colony-forming ability of cells was decreased after taurocyamine treatment. Representative images and quantification from three independent experiments are included. Bars, SD; * *p* < 0.05; ** *p* < 0.01.

Emerging evidence indicates that taurine exerts paradoxical roles in cancer: it can promote or suppress tumor growth [25]. To investigate the role of taurine in HCC, we analyzed the level of taurine in liver cancer tissues from metabolomics databases. Interestingly, taurine levels were elevated in tumor tissues (Figure 1A). Taurine can be synthesized endogenously, but its primary source is dietary intake, which is facilitated by the high-affinity taurine transporter (SLC6A6) [26]. We analyzed *SLC6A6* mRNA levels using GEO datasets GSE124535 and GSE77314. Both datasets revealed significantly elevated *SLC6A6* mRNA levels in liver cancer compared to matched adjacent non-tumor tissues (Figure 1B). Furthermore, elevated *SLC6A6* expression was associated with a poorer prognosis (Figure 1C).

To elucidate the role of taurine in HCC, we treated the human HCC cell line HepG2 with taurine. The results showed that taurine promoted HCC cell proliferation after 48 h (Figure 1D,E). Consistent with these results, colony formation assays confirmed that taurine increased the cells’ ability to form colonies (Figure 1F). To further examine the function of taurine in HCC, we were prompted to test whether a taurine transporter inhibitor can inhibit cell proliferation. Taurocyamine, an inhibitor of taurine transmembrane transport, has been validated in both in vivo and in vitro [27,28]. The effect of taurocyamine on HepG2 proliferation was evaluated. The results revealed that taurocyamine effectively inhibited proliferation rates (Figure 1G–I). Together, these results indicate the crucial role of taurine in promoting HCC cell proliferation.

### 3.2. Taurine Promotes c-MYC/AKT/shp53-Induced Hepatocellular Carcinoma

**Figure 2:**
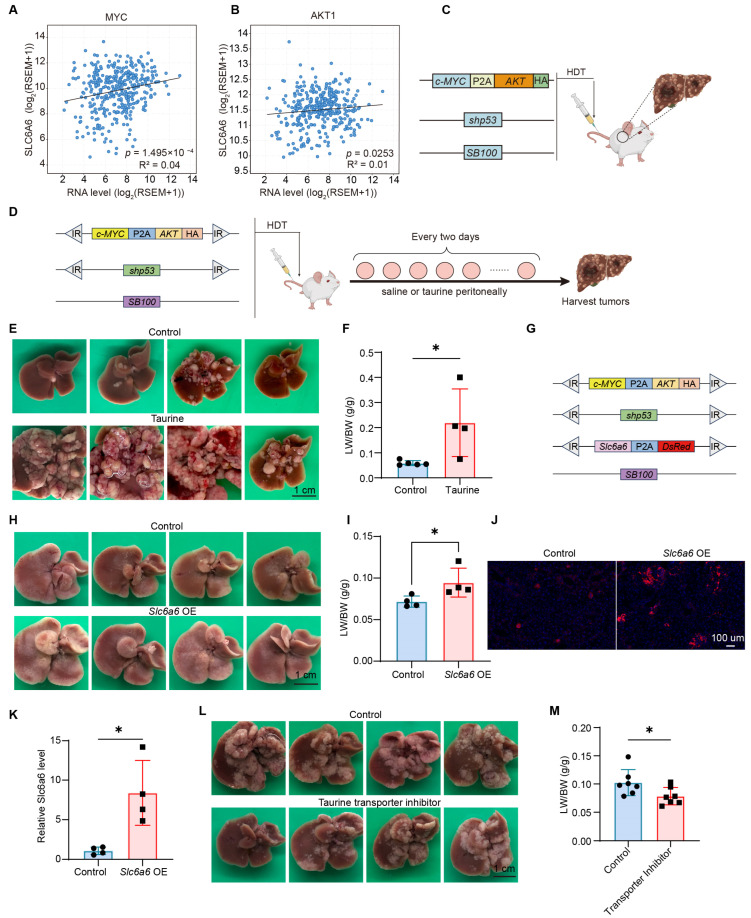
Taurine impacts *c-MYC*/*AKT*/*shp53*-driven hepatocarcinogenesis. (**A**) Correlations between hepatic *c-MY*C and *SLC6A6* expression in patients (TCGA) were calculated by Pearson’s correlation. (**B**) Correlations between hepatic *AKT* and *SLC6A6* expression in patients (TCGA) were calculated by Pearson’s correlation. (**C**) Schematic of plasmids used for HDT. (**D**) Schematic of plasmids used for HDT and intraperitoneal injection. (**E**,**F**) Representative liver images and Liver-to-body weight ratio (LW/BW) quantification from mice intraperitoneally injected with normal saline or taurine (control: *n* = 5; taurine: *n* = 4). (**G**–**K**) Schematic of plasmids used for HDT, Representative liver images, LW/BW ratio, DsRed protein expression, qPCR detection of *Slc6a6* expression from mice after injection of control or *Slc6a6*-overexpressing plasmids (*n* = 4). (**L**,**M**) Representative liver images and LW/BW ratio quantification from mice injected with normal saline or Guanidinoethyl sulfonate (*n* = 7). Bars, SD; * *p* < 0.05.

Hyperactivation of the WNT/β-catenin pathway is a major contributor to the HCC, as one of the target genes of the WNT/β-catenin signaling pathway, MYC is aberrantly activated in patients with HCC [2]. Furthermore, studies have shown that the AKT pathway is activated in 50% of HCC cases [29]. Interestingly, in HCC patients, there is a positive correlation between the expression level of the *SLC6A6* gene and those of the *c-MYC* and *AKT* genes (Figure 2A,B). Based on the correlation between *SLC6A6*, *c-MYC*, and *AKT*, we used the Sleeping Beauty transposon and hydrodynamic tail vein injection system (HDT) to deliver *c-MYC*, *AKT*, and *shp53* (Figure 2C), which have been characterized to induce HCC [30], and then utilized this model to explore the role of taurine in HCC in vivo.

To investigate if high levels of taurine are critical for HCC, we hydrodynamically injected 6-week-old mice with transposon plasmids encoding *c-MYC*, *AKT*, and *shp53*; the mice then received an intraperitoneal injection of taurine (Figure 2D). The results showed that administration of taurine significantly promoted the formation of *c-MYC*/*AKT*/*shp53*-driven HCC (Figure 2E), as evidenced by an elevated liver-to-body weight ratio (LW/BW) (Figure 2F). Furthermore, overexpression of *Slc6a6* also increased tumor burden and elevated the LW/BW ratio (Figure 2G–I). Fluorescence and qPCR confirmed the expression of *Slc6a6* (Figure 2J,K). To further determine the pro-tumorigenic effect of taurine, six-week-old mice were treated with taurocyamine intraperitoneally (200 mg/kg) every two days to block the transport of taurine. Taurocyamine-treated mice livers exhibited less tumor burden and a lighter LW/BW ratio compared to vehicle-treated mice (Figure 2L,M). In summary, these data demonstrate that taurine dramatically promotes HCC formation.

### 3.3. Taurine Enhanced the Level of Bile Acids

**Figure 3:**
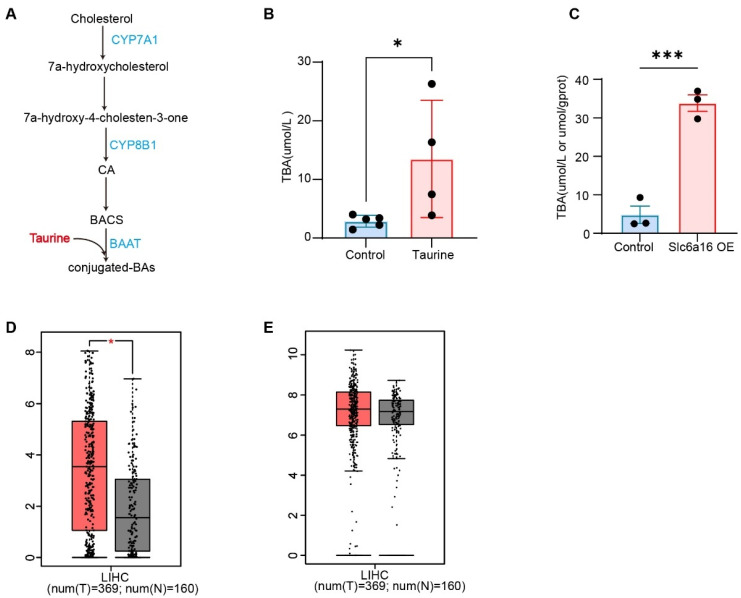
Taurine enhanced the level of bile acids. (**A**) Schematic of bile acid synthesis: primary bile acids (BA) are synthesized from cholesterol by the classic pathway or the alternative pathway. BA-CoA: amino acid N-acyltransferase (BAAT) then catalyse BA conjugation with glycine or taurine to form bile salts. (**B**) Total BA levels in the serum of mice intraperitoneally injected with normal saline or taurine (Taurine, i.p., 100 mg/kg, every other day; Control: *n* = 5; taurine: *n* = 4). (**C**) Total BA levels in the serum of mice with *Slc6a6* overexpression (*n* = 3). (**D**) Box plot of the expression of *CYP7A1* in HCC tissues and adjacent normal tissues (red represents tumor tissues, gray represents normal tissues). (**E**) Box plot of the expression of *BAAT* in HCC tissues and adjacent normal tissues (red represents tumor tissues, gray represents normal tissues). Bars, SD; * *p* < 0.05; *** *p* < 0.001.

In the liver, the primary role of taurine is to serve as a substrate for BA synthesis. BA biosynthesis is initiated by cholesterol 7α-hydroxylase (CYP7A1). The products are the primary BAs, and taurine or glycine conjugates with primary BAs in the liver via BAAT to form bile salts (Figure 3A). To investigate the role of taurine downstream in HCC formation, we tested the levels of BAs in mice that received an intraperitoneal injection of taurine or overexpressed *Slc6a6*. Serum total BAs levels were significantly elevated in taurine-treated mice and mice with *Slc6a6* overexpression (Figure 3B,C). Furthermore, the expression levels of *BAAT* and *CYP7A1* are increased in tumor tissues compared with non-tumor tissues (Figure 3D,E). Collectively, these results indicate that taurine is essential for BAs synthesis and may promote HCC by BAs.

### 3.4. BAs Are Required for the Formation of Hepatocellular Carcinoma Induced by Taurine Administration

**Figure 4:**
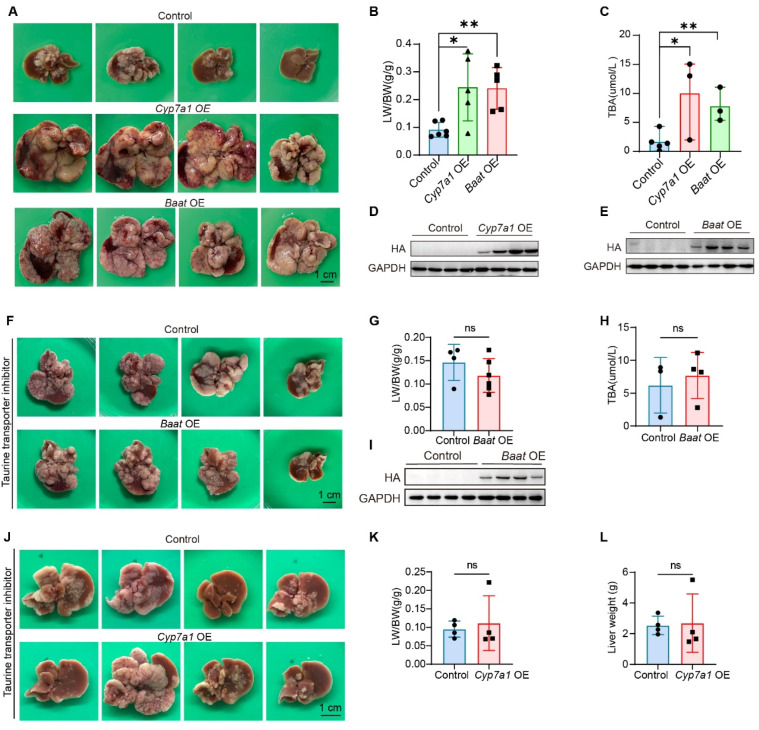
The BAs synthesis pathway is a key downstream effector of taurine-induced HCC. (**A**–**E**) Representative liver images, LW/BW ratio, total BA levels, Western blot verification of HA-tagged *Cyp7a1*, HA-tagged *Baat* overexpression (GAPDH: loading control) from mice injected with control plasmids, *Cyp7a1*-overexpressing plasmids, or *Baat*-overexpressing plasmids (control: *n* = 6, Cyp7a1 OE: *n* = 5, Baat OE: *n* = 5). (**F**–**I**) Representative liver images, LW/BW ratio, total BA levels, Western blot verification of HA-tagged *Baat* overexpression (GAPDH: loading control) from mice injected with control plasmids, or *Baat*-overexpressing plasmids, which were administered taurine intraperitoneally every two days (control: *n* = 4, Baat OE: *n* = 6). (**J**–**L**) Representative liver images, LW/BW ratio, liver weight from mice injected with control plasmids, or *Cyp7a1*-overexpressing plasmids, which were administered taurine intraperitoneally every two days (*n* = 4). Bars, SD; * *p* < 0.05; ** *p* < 0.01; ns *p* > 0.05.

To investigate the functional role of BAs in HCC, we overexpressed the *Cyp7a1* gene, which encodes the first enzyme in bile acid synthesis. Our results demonstrated that *Cyp7a1* overexpression significantly increased tumor burden in the *c-MYC*/*AKT*/*shp53* mouse HCC model (Figure 4A,B). Furthermore, *Baat* overexpression also significantly elevated tumor burden and LW/BW ratio (Figure 4A,B). Importantly, overexpression of *Cyp7a1* and *Baat* markedly increased total BA levels (Figure 4C). Western blot confirmed the expression of *Cyp7a1* and *Baat* (Figure 4D,E). This finding aligns with a recent report that BAs promote liver cancer [23]. To further validate the relevance between taurine and bile acids in HCC, six-week-old mice were injected with *Baat* or *Cyp7a1*-overexpressing plasmids via HDT and administered taurocyamine intraperitoneally every two days. Taurocyamine treatment can effectively block the pro-tumorigenic effect and the decreased BAs levels induced by *Baat* (Figure 4F–I) or *Cyp7a1* overexpression (Figure 4J–L). Collectively, the obtained results indicate that taurine promotes HCC through the BAs pathway.

### 3.5. Bile Acids Regulate Cell Cycle, p53 Signaling Pathway and Metabolic Pathways

**Figure 5:**
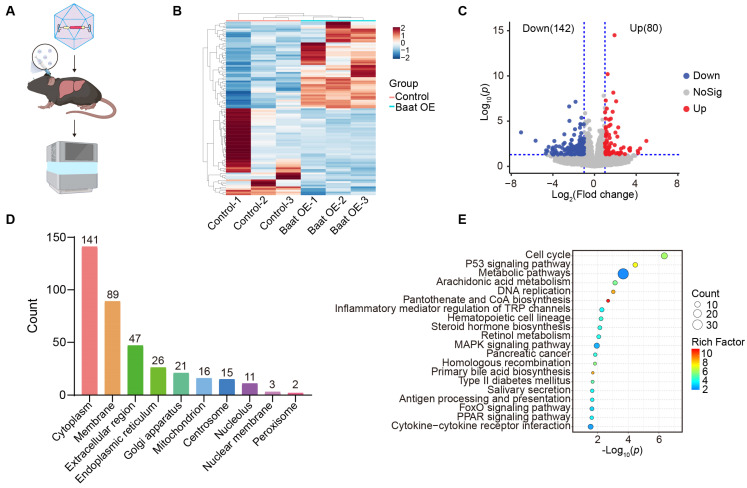
*Baat* overexpression regulates cell cycle, p53 signaling pathway and metabolic pathway. (**A**) Schematic diagram illustrating the sample preparation process for RNA sequencing (RNA-seq). (**B**) Hierarchical clustering heatmap of differentially expressed genes in *Baat* overexpression (*Baat* OE) versus control (NC) mice livers. (**C**) Volcano plot depicting enriched DEGs identified from RNA-seq data. (**D**) Subcellular localization of DEGs based on GO cellular component analysis. (**E**) KEGG pathway enrichment analysis of DEGs in the livers of *Baat*-overexpression mice.

To investigate the role of the downstream pathway of bile acids in HCC development, we performed RNA sequencing (RNA-seq) analysis on liver tissues from mice harvested two days after injection with adenoviral GFP or adenoviral Baat (Figure 5A). Hierarchical clustering of differential gene levels showed distinct patterns between the *Baat*-overexpressing and control groups (Figure 5B). Differential gene expression and enrichment analyses were performed. A total of 222 differentially expressed genes (DEGs) were identified, with 80 genes upregulated and 142 genes downregulated in Baat-overexpressing mice compared with control mice (Figure 5C, Supplementary Table S1). Subcellular localization analysis of DEGs indicates their primary distribution in the cytoplasm, membrane and extracellular region (Figure 5D). Enrichment analysis of the DEGs highlighted significant Gene Ontology (GO) terms, including cell cycle, p53 signaling pathway and metabolic pathways (Figure 5E). These findings underscore the regulatory function of BAs in essential cancer-related signaling pathways, indicating their involvement in modulating cell proliferation and metabolism during HCC formation.

## 4. Discussion

HCC is a leading cause of cancer-related mortality worldwide [31,32]. A broad range of treatment options is available for patients with HCC [33]. However, these treatments have only achieved limited clinical benefits. Therefore, there is an urgent need to explore and understand the underlying molecular mechanisms of HCC formation for the purpose of identifying prospective therapeutic targets. Our results show that liver cancers display enhanced taurine levels compared to normal livers. Taurine supplementation can promote the formation of HCC by promoting BA synthesis. Our study may provide a novel therapeutic target for HCC.

Taurine is a sulfur-containing, non-proteinogenic amino acid that is localized in the heart, liver, and skeletal muscle [34]. The levels of taurine are maintained by exogenous uptake and endogenous synthesis. In the endogenous synthesis pathway, cysteine dioxygenase-1 converts cysteine to cysteine sulfinic acid [35], which is then decarboxylated by cysteine sulfinic acid decarboxylase to form hypotaurine, which is subsequently oxidized by flavin-containing monooxygenase 1 to produce taurine [36]. However, tumors rely on the uptake of exogenous, rather than on endogenous synthesis [37]. Therefore, we overexpressed the taurine transporter to further investigate the role of taurine in HCC. This is consistent with our findings that inhibition of the taurine transporter suppresses HCC formation. Taken together with previous studies showing that knockout of *Slc6a6* induces taurine depletion in a variety of tissues [38], our work highlights the potential harm of taurine supplementation in HCC patients.

There are multiple pathways for exogenous taurine intake because it is a common component in foods and energy drinks. The Food and Drug Administration (FDA) classifies taurine as a Generally Recognized as Safe (GRAS) substance, authorizing its commercial application in dietary supplements and beverage products. Commercial energy drinks contain taurine at concentrations around 4000 mg/L [39]; by contrast, dietary sources generally provide less than 800 mg per 100 g [17]. According to an assessment by Intertek Scientific & Regulatory Consultancy, an omnivorous diet provides approximately 9–400 mg of taurine per person per day, and a daily intake of 3000 mg is proposed as a benchmark for evaluating human dietary exposure to taurine (https://www.fda.gov/food/gras-notice-inventory/agency-response-letter-gras-notice-no-grn-000586; (accessed on 9 December 2025)). Accumulating evidence indicates that taurine contributes to neuroprotection, reduces chemotherapy-related toxicity [40], and promotes anti-tumor immune functions [20]. However, in this study, we found that taurine supplementation at a dose of 100 mg/kg promoted hepatocellular carcinoma progression in mice. Additionally, a study on leukemia urges caution in considering taurine supplementation [4]. Interestingly, both low-dose taurine (25 mg/kg–50 mg/kg) and taurine-containing drinks at equivalent doses can significantly promote lung tumor growth, yet this promotive effect attenuates with increasing taurine dosage [41]. This correlation underscores that dosage may be a key determinant of taurine’s functional effects and highlights the need to restrict excessive consumption of energy drinks. Future research should further investigate the favorable intake concentrations of taurine in different contexts or establish its maximum safe allowable levels in humans.

Taurine has been demonstrated to play a crucial role in the survival of many cancer cells [4,20]. However, it exerts complex roles in cancer: it can enhance antitumor immunity or promote tumor growth. Immune cells use taurine to enhance cytokine production and cytotoxin activity [20,25,42], while tumor cells use taurine to fuel metabolism [4]. In the liver, although the *Slc6a6* gene is highly expressed in macrophages, the most well-established role of taurine is its conjugation with bile acids for subsequent excretion into bile [43,44]. This is consistent with our findings that taurine supplementation promotes the production of bile acids. In addition, inhibition of taurine transport can effectively block the pro-tumorigenic effect and the decrease in BA levels induced by *Baat* or *Cyp7a1.*

The accumulation of BAs is a metabolic feature of HCC [45]. Emerging evidence shows that bile acids are associated with immunity [23,46,47]. For instance, knockout of *SLC7A1* and *BAAT* inhibits tumor-specific T cell responses and enhances tumor growth [23]. Furthermore, the influences of microbial-derived BAs in regulating natural killer T cell responses to liver cancer have been described [48]. Here, overexpression of *Cyp7a1* and *Baat* promotes *c-MYC*/*AKT*/*shp53*-driven HCC and regulates cell cycle, p53 signaling pathway and metabolic pathways. Our study provides a non-immunological pathway through which bile acids promote tumorigenesis. However, the specific mechanisms remain to be elucidated. Future studies need to investigate which signaling pathway is regulated by the BAs. Furthermore, we only overexpressed *Cyp7a1* and *Baat* and failed to knock out these two genes. Subsequent studies with knockout mice are recommended to assess their effects on taurine and downstream signaling pathways.

## 5. Conclusions

Our study reveals that taurine levels are increased in HCC. These high levels of taurine promote the formation of HCC in vivo and vitro. Importantly, increased taurine availability enhanced BAs biosynthesis, and the accumulation of BAs further regulates cell cycle, p53 signaling pathway and metabolic pathways. These findings indicate that the taurine/bile acid axis is a crucial therapeutic target for HCC, offering promising prospects for the treatment of this disease.

## Data Availability

The original contributions presented in this study are included in the article. Further inquiries can be directed to the corresponding author.

## Supplementary Materials

The following supporting information can be downloaded at: https://www.mdpi.com/article/10.3390/metabo16010006/s1, Table S1: *Baat* differentially expressed genes.

## Abbreviations

The following abbreviations are used in this manuscript:

BAs	bile acids
HCC	hepatocellular carcinoma
HDT	hydrodynamic tail vein injection
CYP7A1	cholesterol 7α-hydroxylase
BAAT	Bile Acid-CoA: Amino Acid N-Acyltransferase
SLC6A6	Solute Carrier Family 6 Member 6
GO	Gene Ontology
DEGs	Differentially expressed genes
